# An mDia2/ROCK Signaling Axis Regulates Invasive Egress from Epithelial Ovarian Cancer Spheroids

**DOI:** 10.1371/journal.pone.0090371

**Published:** 2014-02-28

**Authors:** Krista M. Pettee, Kaitlyn M. Dvorak, Andrea L. Nestor-Kalinoski, Kathryn M. Eisenmann

**Affiliations:** 1 Department of Biochemistry and Cancer Biology, University of Toledo Health Science Campus, Toledo, Ohio, United States of America; 2 Department of Surgery, University of Toledo Health Science Campus, Toledo, Ohio, United States of America; Karolinska Institutet, Sweden

## Abstract

Multi-cellular spheroids are enriched in ascites of epithelial ovarian cancer (OvCa) patients. They represent an invasive and chemoresistant cellular population fundamental to metastatic dissemination. The molecular mechanisms triggering single cell invasive egress from spheroids remain enigmatic. mDia formins are Rho GTPase effectors that are key regulators of F-actin cytoskeletal dynamics. We hypothesized that mDia2-driven F-actin dynamics promote single cell invasive transitions in clinically relevant three-dimensional (3D) OvCa spheroids. The current study is a dissection of the contribution of the F-actin assembly factor mDia2 formin in invasive transitions and using a clinically relevant ovarian cancer spheroid model. We show that RhoA-directed mDia2 activity is required for tight spheroid organization, and enrichment of mDia2 in the invasive cellular protrusions of collagen-embedded OVCA429 spheroids. Depleting mDia2 in ES-2 spheroids enhanced invasive dissemination of single amoeboid-shaped cells. This contrasts with spheroids treated with control siRNA, where a mesenchymal invasion program predominated. Inhibition of another RhoA effector, ROCK, had no impact on ES-2 spheroid formation but dramatically inhibited spheroid invasion through induction of a highly elongated morphology. Concurrent inhibition of ROCK and mDia2 blocked single cell invasion from ES-2 spheroids more effectively than inhibition of either protein alone, indicating that invasive egress of amoeboid cells from mDia2-depleted spheroids is ROCK-dependent. Our findings indicate that multiple GTPase effectors must be suppressed in order to fully block invasive egress from ovarian cancer spheroids. Furthermore, tightly regulated interplay between ROCK and mDia2 signaling pathways dictates the invasive capacities and the type of invasion program utilized by motile spheroid-derived ovarian cancer cells. As loss of the gene encoding mDia2, *DRF3,* has been linked to cancer progression and metastasis, our results set the stage for understanding molecular mechanisms involved in mDia2-dependent egress of invasive cells from primary epithelial tumors.

## Introduction

Ovarian cancer (OvCa) is the 5^th^ leading cause of cancer-related deaths in American women. The American Cancer Society predicts 22,000 new diagnoses and 15,000 deaths from epithelial ovarian cancer (EOC) in 2012. Cells derived from the ovarian surface epithelium account for >90% of primary OvCa and metastatic lesions [1]. Exfoliated OvCa cells are detected in peritoneal ascites as single cells, small aggregates or highly-ordered and compacted multi-cellular spheroids [1]–[5]. Compacted spheroids are poorly understood invasive and chemoresistant structures. Cell-cell adhesions, supported in part by N- and E-cadherin drive the formation and compaction of OvCa spheroids [6]. It is unclear what specific molecular or environmental cues contribute to invasive transitions in the spheroids.

A growing body of evidence suggests that compact spheroids promote OvCa metastasis within the peritoneal cavity. *In vitro,* cells within OvCa spheroids undergo anchorage-independent growth, disaggregate upon adhering to three-dimensional (3D) collagen-I gels and invade underlying mesothelial cells [7]–[9]. Importantly, spheroid formation and compaction directly correlate with invasion [3], [4]. Human OvCa cells with the capacity to form compact spheroids *in vitro* can generate robust tumors when injected intraperitoneally into immunodeficient mice. In contrast, non-spheroid forming cells fail to form tumors in mice [10]. The apparent connection between the formation of compact spheroids and dissemination of invasive OvCa cells underscores the need for better understanding of molecular mechanisms supporting these processes [11].

In response to extracellular cues, epithelial-derived cancer cells can convert to a mesenchymal-like phenotype. This can be achieved via dissociation of tight and adherens junctions (AJ), detachment of single cells from epithelial sheets or 3D structures, and migration into adjacent tissues (reviewed in [12]–[15]). Hallmark cellular and molecular changes associated with the epithelial-mesenchymal transition (EMT) and invasion into the surrounding stroma include loss of E-cadherin expression, upregulation of matrix metalloprotease (MMP), activation of transcriptional regulators and adoption of a spindle-like, polarized fibroblastic morphology. During mesenchymal migration, expansion of the leading edge and retraction of the trailing edge of the motile cells involves coordinate regulation of cortical F-actin polymerization and actomyosin contraction. F-actin assembly precedes contraction to promote extension of actin-rich protrusions, and ROCK-mediated phosphorylation of myosin light chain (pMLC) regulates actomyosin contraction [16].

Formins have emerged as key regulators of F-actin and microtubule cytoskeletal dynamics. Formin family proteins are Rho GTPase effectors. The mammalian Diaphanous (mDia)-related formins (mDia1-3) have critical roles in diverse cellular activities, including gene transcription, cell cycle progression, and membrane trafficking. mDia proteins also regulate F-actin networks critical for maintaining the integrity of multi-cellular epithelial structures [17]–[22]. For example, depletion of mDia inhibits the formation of cell-cell junctions in MDCK cells [23], and disrupts MCF-7 monolayers by mislocalizing E-cadherin away from cell-cell contacts [24]. Furthermore, suppressing Diaphanous, the mDia homologue in *D. melanogaster,* decreased pMLC at AJs and destabilizes normal tissue boundaries and increased cell protrusiveness [25]. Collectively, these data implicate the mDia-Rho signaling axis in the organization of complex 3D structures important for morphogenesis, and potentially, for tumor progression.

Several lines of evidence suggest that formins play conserved roles in regulating cancer cell motility, invasion and metastasis. mDia2 influences the invasive behavior of breast cancer cells, which ultimately depends on MMP and formation of actin-rich protrusions called invadopodia [26]. Interaction of mDia2 with its negative regulator, DIP [27], promotes the transition of mesenchymal MDA-MB-231 cells into highly motile and invasive ROCK-dependent amoeboid cells [28]. Similarly, modulation of mDia2 also affects amoeboid invasion and migration in DU145 prostate cancer cells [29], [30]. A related formin, mDia1 directs neutrophil chemotaxis [31], [32], cell invasion [33], and T-cell migration and trafficking into peripheral lymphoid organs [34], [35]. Other formin family members, including isoforms of FRL, FHOD and Formin1, have also been implicated in regulating migratory plasticity and interconversion between mesenchymal and amoeboid motility in breast and other tumor cells[36]–[41].

The genes encoding mDia or FMNL/FRL are frequently lost in breast, prostate, colorectal, hepatic carcinomas, and hematopoetic cancers [29], [30], [41], [42]. mDia proteins also function in the normal ovary as well as ovarian cancers. Disruption of *Diaphanous* is associated with premature ovarian failure [43]. Loss of *DIAPH1/DRF1*, the gene encoding mDia1, was shown in a model of OvCa progression [10]. Furthermore, the genes encoding mDia2 (*DIAPH3/DRF3*) and formin 1 (*FMN1*) were downregulated in early to late stage OvCa cells. This was accompanied by disruption in F-actin accumulation and increases in cell deformability [44], [45]. Taken together, the foregoing observations suggest a role for mDia-controlled actin dynamics in progression of ovarian disease. It is unclear if changes in the activation state of mDia influence cell-cell adhesions to maintain OvCa spheroid integrity, or if modulation of mDia directs the transitions that underlie cell invasion into the underlying mesothelium.

To explore these questions, we examined the role of Rho/mDia signaling in OvCa migration and invasion. Our results show that a RhoA/mDia2/ROCK signaling axis drives invasive transitions in ES-2 OvCa cells in 2D cultures and a 3D spheroid invasion model. Activation of RhoA was increased during spheroid formation, while suppression of mDia2 disrupted spheroid architecture. In contrast to the effects of mDia2, depletion of mDia1 had no effect on the formation or integrity of spheroids. When mDia2-depleted spheroids were embedded into collagen-I gels, it was possible to enhance single cell invasion through a ROCK-dependent amoeboid transition. These findings demonstrate the interdependence of mDia2 and ROCK for the promotion of invasive transitions in OvCa spheroids.

## Results

### Spatial localization of mDia proteins in migrating human OVCA cells

**Figure 1:**
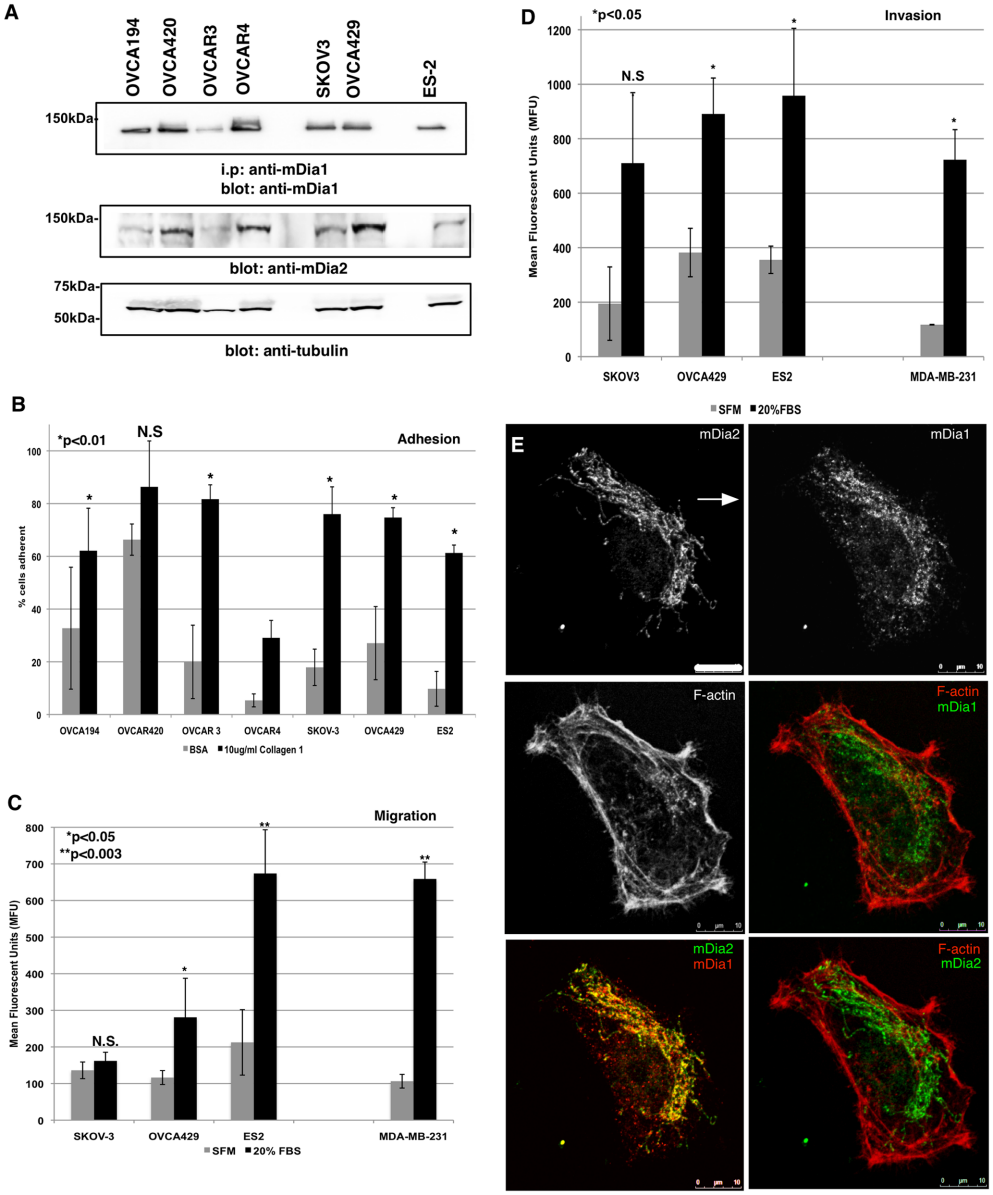
Polarized mDia expression in migrating OvCa cells. A. mDia1 and mDia2 were assessed by immunoprecipitation and/or Western blotting in a panel of human OvCa cells. Tubulin was blotted as a loading control. B. Cell adhesion to BSA- or Collagen-I-coated wells. The experiment was performed in triplicate wells and the experiment was repeated thrice. p values are BSA vs. collagen-I for each cell line. C. Transwell migration assays were performed in triplicate wells and the experiment was repeated thrice for 5 h towards SFM or 20% FBS. MDA-MB-231 human breast cancer cells are a positive migration control. p values are for SFM vs. 20% FBS for each cell line. D. Transwell invasion assays were performed in triplicate wells and the experiment was repeated thrice for 24 h through 2 mg/ml Collagen-I gels towards either SFM or 20% FBS. MDA-MB-231 cells are a positive invasion control. p values are SFM vs. 20% FBS for each cell line. E. OVCA429 cells migrated towards 20% FBS for 5 h in a Dunn chamber. mDia1, mDia2 and the F-actin architecture were visualized by IF. Arrow denotes direction of the gradient. N.S. =  not significant. All error bars correspond to SDs Shown are representative experiments from each assay.

We obtained a panel of human OvCa cell lines representing several clinical subtypes, including serous and clear cell adenocarcinomas. Some of these cell lines form compact spheroids (OVCA429 and ES-2) while others form looser honeycomb structures (SKOV3) (Figure S1 and [4]). All of the cell types expressed mDia1 and mDia2, regardless of spheroid-forming ability (Figure 1A).

The OVCA panel was screened for adhesive, migratory and invasive properties (Figure 1, B–D, respectively) to identify a highly invasive, spheroid-forming cell line. SKOV3, OVCA429 and ES-2 cells were equally adhesive on collagen 2D substrates. However, the OVCA429 and ES-2 cells were more migratory and invasive than the SKOV-3 line in response to FBS gradients. Therefore, our remaining studies were conducted with either OVCA429 or ES-2 cells.

The spatial localization of endogenous mDia proteins was evaluated in migrating OVCA429 cells using a Dunn chamber to maintain a stable FBS gradient. Immunofluorescence (IF) was performed after 5 h migration (Figure 1E). Both mDia1 and mDia2 exhibited a polarized distribution oriented toward the FBS gradient within migrating OVCA429 cells (arrow indicates gradient location). mDia1 and mDia2 localized predominantly to the lamellae, as opposed to the F-actin rich lamellapodia, as previously seen in migrating epithelial cells [46]. These results suggest a possible role for mDia1 and/or mDia2 in stabilizing cortical actin pools during OvCa chemotaxis.

### Requirement for mDia2 in chemotactic migration in OVCA429 cells

**Figure 2:**
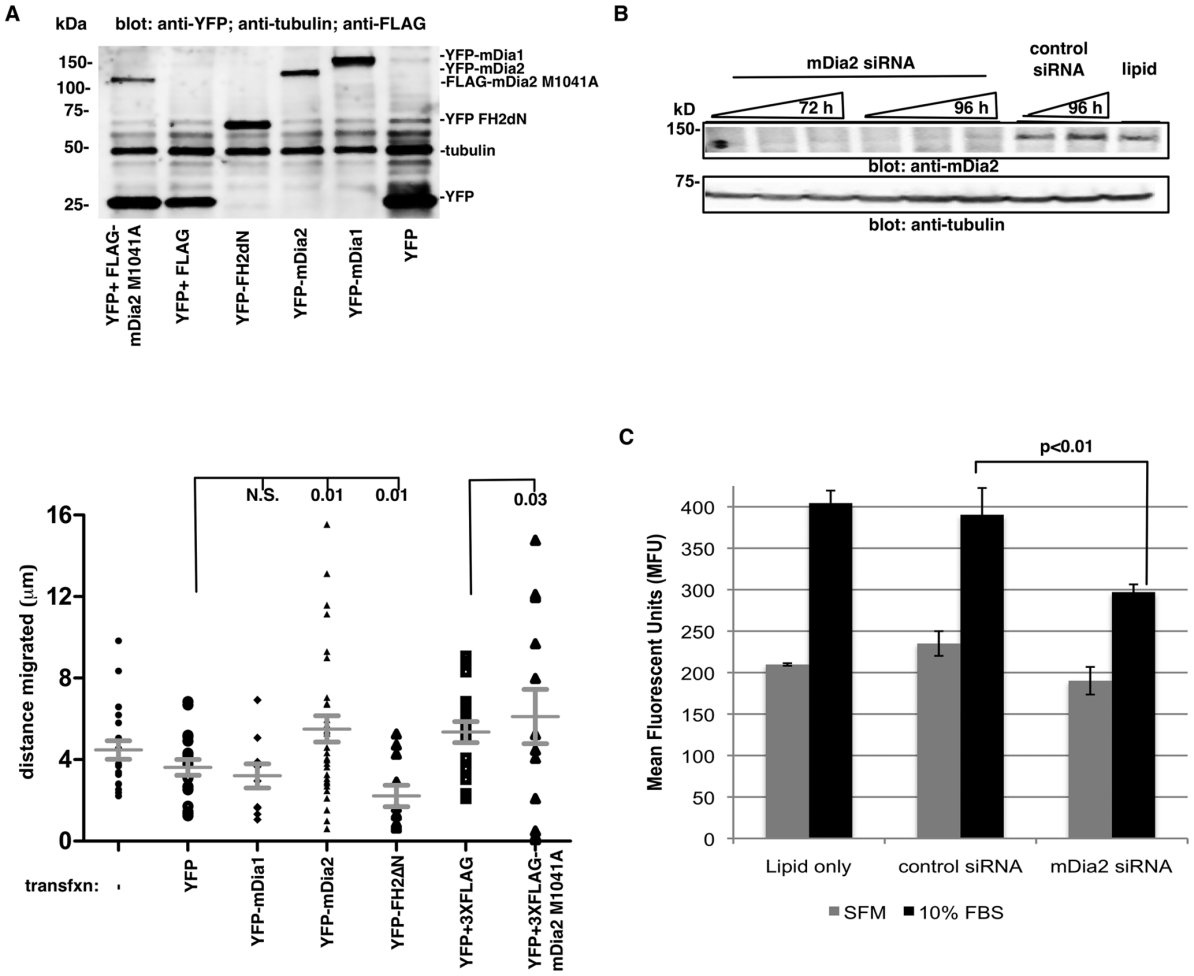
A role for mDia2 in chemotactic migration in OVCA429 cells. A. Transfected OVCA429 migrated for 5% FBS. Live single-cell tracking of transfected cells was performed, and total distance migrated calculated. At least 10 cells were tracked per condition. p values listed are calculated relative to respective empty vector controls. B. OVCA429 cells were transfected with lipid only, control siRNA or siRNA directed against mDia2 at increasing concentrations (25, 50, 100 nM, denoted by wedges) for either 72 or 96 h. mDia2 depletion was confirmed by Western blot. C. OVCA429 cells were transfected with 100 nM siRNA for 72 h, and transwell migration assays performed for 5 h towards a SFM or 10% FBS. The assay was performed in triplicate wells and the experiment was repeated thrice. A representative experiment is shown. p value shown are 10% FBS treated control vs. mDia2-depleted cells. All error bars correspond to SDs for that representative experiment.

We next asked if mDia protein activity and/or expression were required for chemotactic migration. The distance migrated by transfected OVCA429 cells in a Dunn chamber was quantified by live-cell tracking. Cells were engineered to express either mDia-YFP-fusion proteins alone, or various combinations of control YFP vector and FLAG-tagged proteins. mDia overexpression was validated by Western blotting (Figure 2A). Cells expressing dominant-negative YFP-mDia-FH2ΔN, which interferes with both mDia1 and mDia2-driven F-actin assembly, [34], [47], [48] migrated poorly relative to YFP controls. Conversely, cells expressing the constitutively-activated mDia2 M1041A [49], in which the autoinhibited state is relieved, exhibited increased migration relative to YFP+3XFLAG empty vector controls. mDia1 overexpression did not alter cell migration relative to empty vector controls. These data suggest that mDia2 autoinhibition must be released in order for effective cell migration to occur. In the next series of studies, the role of mDia2 in chemotactic migration was assessed by seeding mDia2-depleted OVCA429 cells into transwells. While lipid and control siRNA-treated cells robustly migrated toward FBS (relative to serum-free control wells), migration was significantly reduced in the mDia2-depleted cells (Figure 2C). Collectively, these data indicate that mDia2 expression and/or activity is required for optimal chemotactic migration of OVCA429 cells.

### The RhoA/mDia2 signaling axis in formation of compact OvCa spheroids

**Figure 3:**
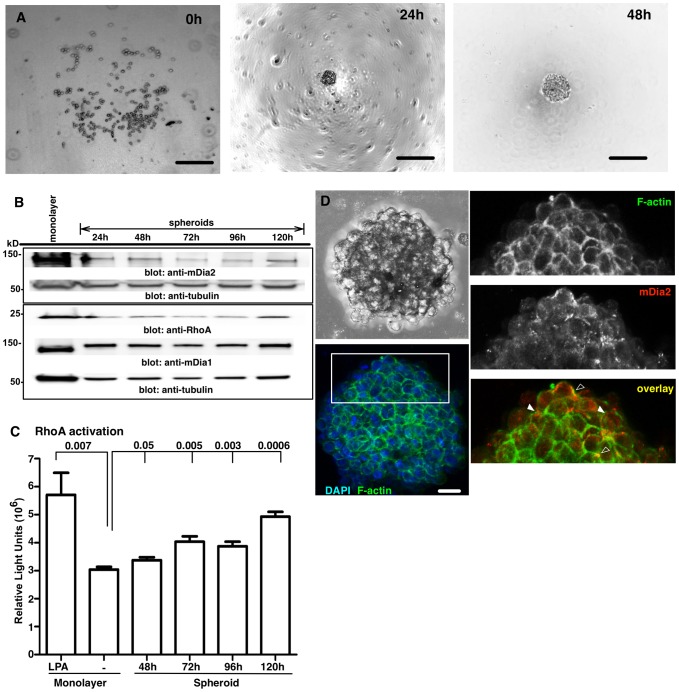
Expression of the RhoA:mDia axis in compacted ES-2 spheroids. A. ES-2 spheroids imaged by brightfield microscopy. Scale bar =  250 µm. (Note: out of focus plate-coating imaging artifact and/or condensation within irregular plastic plates seen here is common to BF imaging). B. Lysates from ES-2 monolayers or spheroids were Western-blotted after the designated times. C. RhoA G-LISAs were performed upon untreated or LPA-treated ES-2 monolayers, or spheroids. p values are relative to untreated monolayer. The experiment was performed in quadruplicate wells and the experiment was then repeated thrice. Data combined from 3 experiments are shown. p values are shown above bars and are relative to untreated monolayer controls. D. Spheroids formed for 72 h were embedded, fixed and mDia2 visualized by confocal microscopy. Images were obtained using a 20× objective with 3D-rendering of serial slices. Bar =  25 µm. Open arrows indicate areas of overlap, while closed arrows indicate a juxtaposition of F-actin and mDia2. All error bars correspond to SDs for that representative experiment, unless noted.

Rho-responsive mDia proteins can regulate cell-cell contacts and promote single cell motility [18], [26]–[28], [49]. We next determined whether the RhoA/mDia2 signaling axis was involved in OvCa spheroid formation. ES-2 cells rapidly formed highly compacted spheroids (Figure S1) and were used for the remaining studies. The requirement for Rho GTPase activity in spheroid formation was tested by exposing ES-2 cells to increasing concentrations (0.5–2.0 µg/ml) of C3 transferase, an inhibitor of RhoA-C. As shown in Figure S2A, C3-treated spheroids were more loosely organized and less compacted relative to vehicle-treated controls. Since previous work has shown that total levels of RhoA are increased in spheroids derived from hepatocarcinoma [50], we next asked if changes in RhoA activity occur in conjunction with formation of ES-2 spheroids. Spheroids were formed from 500 cells within 24 h (Figure 3A). RhoA and expression of its downstream effectors, mDia1 and mDia2 was then evaluated in lysates from cell monolayers and pooled spheroids harvested between 24–120 h. Overall, mDia2 levels declined in spheroids relative to monolayers. Within the spheroid populations, time-dependent differences in mDia2 expression were observed, with slightly lower levels noted in 72 and 96 h spheroids, relative to 24, 48 and 120 h spheroids (Figures 3B and S3A). In contrast to mDia2, the levels of mDia1 were increased in spheroids relative to monolayers. In the spheroids mDia1 declined slightly from 24 h through 120 h (Figures 3B and S3B). Total RhoA levels also declined in spheroids, relative to monolayers. Yet, RhoA levels dramatically recovered at 120 h of spheroid formation (Figure 3B and S3C). Since, the interaction of RhoA with its downstream effectors (such as mDia or ROCK) is dependent on activation by GTP-binding, we next quantified the levels of active GTP-bound RhoA in spheroids by G-LISA. The amount of RhoA-GTP was significantly increased in spheroids formed for 48–120 h compared to unstimulated ES-2 cell monolayers, approaching that observed in monolayers treated with a known RhoA activator, lysophosphatidic acid (LPA) (Figure 3C). Therefore, overall levels of RhoA activation are enhanced upon spheroid maturation.

As a first step toward determining if RhoA-dependent activation of mDia2 might affect the formation of cell-cell junctions, we next asked if mDia2 and F-actin are co-localized in spheroids at cell-cell junctions (Figure 3D). Spheroids formed for 72 h revealed distinct F-actin-enriched cell-cell junctions, with punctate mDia2 underlying/proximal to these junctions (filled arrowhead). In some junctions, mDia2 appeared to co-localize with F-actin (unfilled arrowhead). This suggests a potential role for mDia2 in regulating F-actin dynamics necessary for development or maintenance of cell-cell junctions.

### mDia2 depletion leads to the formation of smaller, disorganized spheroids

**Figure 4:**
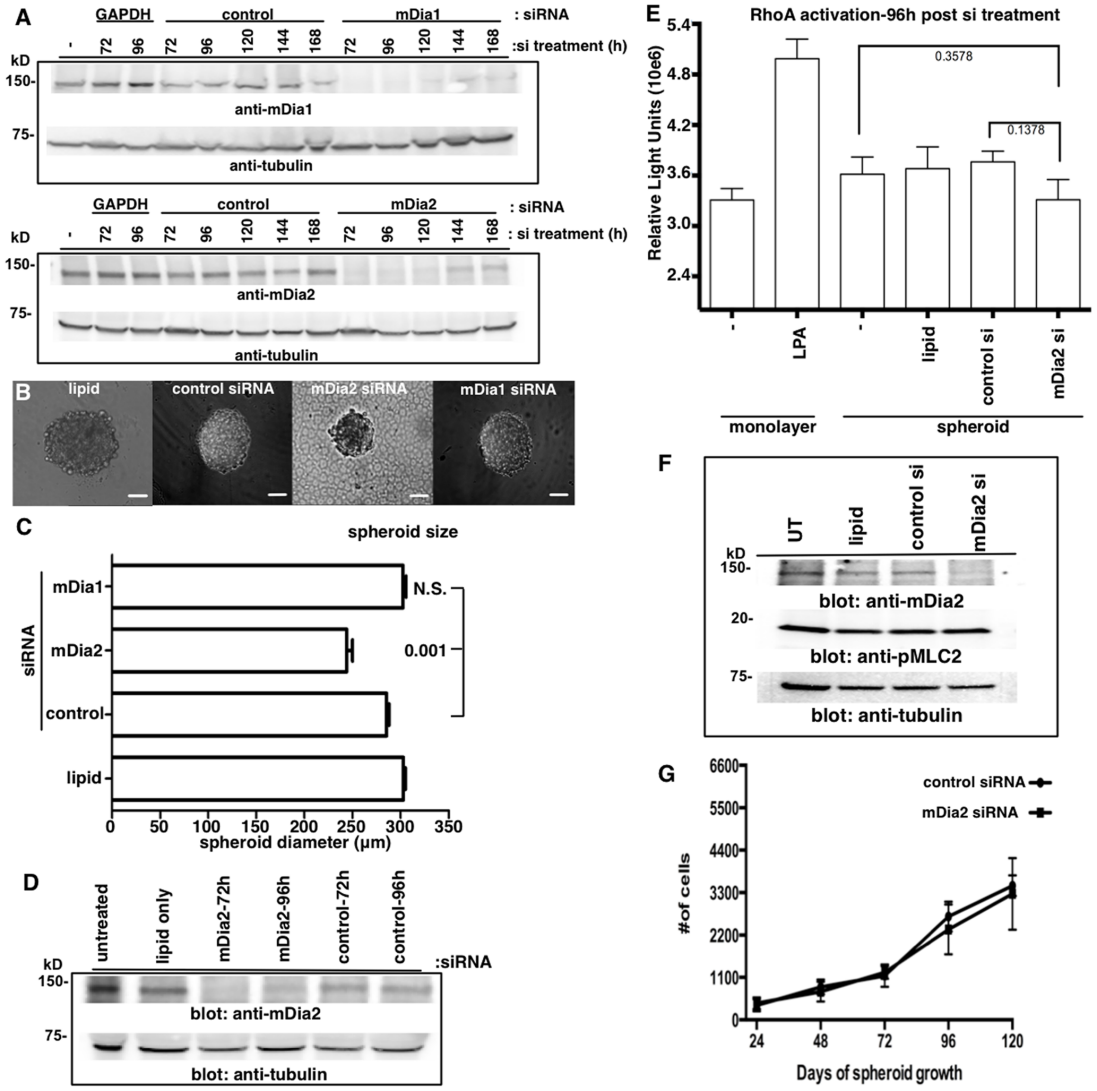
mDia2 depletion induces formation of small, disorganized spheroids. A. siRNA-mediated mDia1 (upper) or mDia2 (lower) knockdowns were performed upon ES-2 monolayers (Note: out of focus plate-coating imaging artifact/condensation within irregular plastic plates seen here is common to BF imaging). B–C. After 72 h post-siRNA or control treatment, spheroids were formed and diameters measured under brightfield microscopy 48 h later (120 h post-siRNA treatment). Bar =  100 µm. At least 30 spheroids were measured for each condition. p values are listed above bars and are relative to control siRNA spheroids. D–E. ES-2 monolayers were untreated or treated with the designated siRNAs for 48 h, spheroids formed for an additional 48 h, and Western blots or RhoA G-LISAs performed upon lysates. LPA is a positive control for RhoA activation. The G-LISA experiment was performed twice, in quadruplicate, and combined data is shown with p values listed to the side of bars. P values are relative to either untreated or control siRNA-treated spheroids, as noted. F. Untreated (UT), lipid only or siRNA-mediated control and mDia2 knockdowns were performed upon ES-2 monolayers for 72 h and pMLC2 levels assessed by Western blotting. G. Control and mDia2 knockdowns were performed for 72 h, and spheroids formed. Spheroids were disaggregated at the indicated times after formation and cells counted. The experiment was repeated 5 times. A representative experiment is shown. Error bars correspond to SDs from a representative experiment.

To further evaluate the role of mDia proteins in ovarian spheroid formation/integrity, we employed an RNA interference strategy. Robust siRNA-mediated knockdown of either mDia2 or mDia1 was validated through 120 h in monolayer cultures, with slight recovery by 144 h (Figure 4A and S4). Spheroids were formed from monolayer cells 72 h post-siRNA treatment. Spheroid diameters were then measured 24–48 h later (96–120 h total siRNA treatment). mDia2 depletion at 48 h resulted in a modest, yet statistically significant decrease (10–20%) in spheroid diameter relative to control siRNA treatment. Similar results were obtained when mDia2 was depleted with siRNA pools or individual siRNA directed against mDia2 (Figure S4), although the suppression of mDia2 expression with pooled siRNA was more consistent and sustained. Therefore, all subsequent studies were performed using pooled siRNA. In addition to the slight decrease in spheroid diameter, mDia2-depleted ES-2 spheroids were visibly less-organized with ragged edges and some single cells loosely attached to the edges. A similar disorganized spheroid phenotype resulted from addition of an mDia1/mDia2 FH2 inhibitor, SMIFH2 (Figure S5) [51]. There results were attributable to mDia2 depletion, as mDia1 depletion had no effect upon spheroid size (Figure 4B,C).

Possible explanations for the slightly smaller size of ES-2 spheroids could include diminished cell proliferation, decreased average cell size, cell detachment or greater spheroid compaction via increased RhoA-directed pMLC2 activity. We therefore determined if RhoA-GTP levels were altered in mDia2-depleted spheroids. mDia2 depletion was validated in spheroids formed for 24–48 h (siRNA treatment for 72–96 h) (Figure 4D). At 96 hrs post-siRNA treatment, RhoA-GTP activity was unaffected in control and mDia2-depleted spheroids (Figure 4E). Consistent with this observation, pMLC2 remained unchanged in cell monolayers (Figure 4F). Furthermore, mDia2 depletion did not change the cellular numbers within disaggregated spheroids (Figure 4G). This indicates that neither cell proliferation nor loss of de-adhered cells was solely responsible for the smaller spheroid size. It also suggests that while the siRNA-mediated depletion of mDia2 causes a more disorganized and irregular spheroid assembly, the remaining mDia2 protein is likely sufficient to sustain both RhoA-mediated contractility and cellular proliferation. The latter concept is consistent with a previous study of mDia1 in T cell activation, where low levels of mDia proteins appeared to be sufficient to sustain some cellular processes (*e.g*., F-actin accumulation vs. microtubule dynamics), while concurrently impairing other functions [52].

### mDia2 depletion enhances ES-2 spheroid invasion via an amoeboid transition

**Figure 5:**
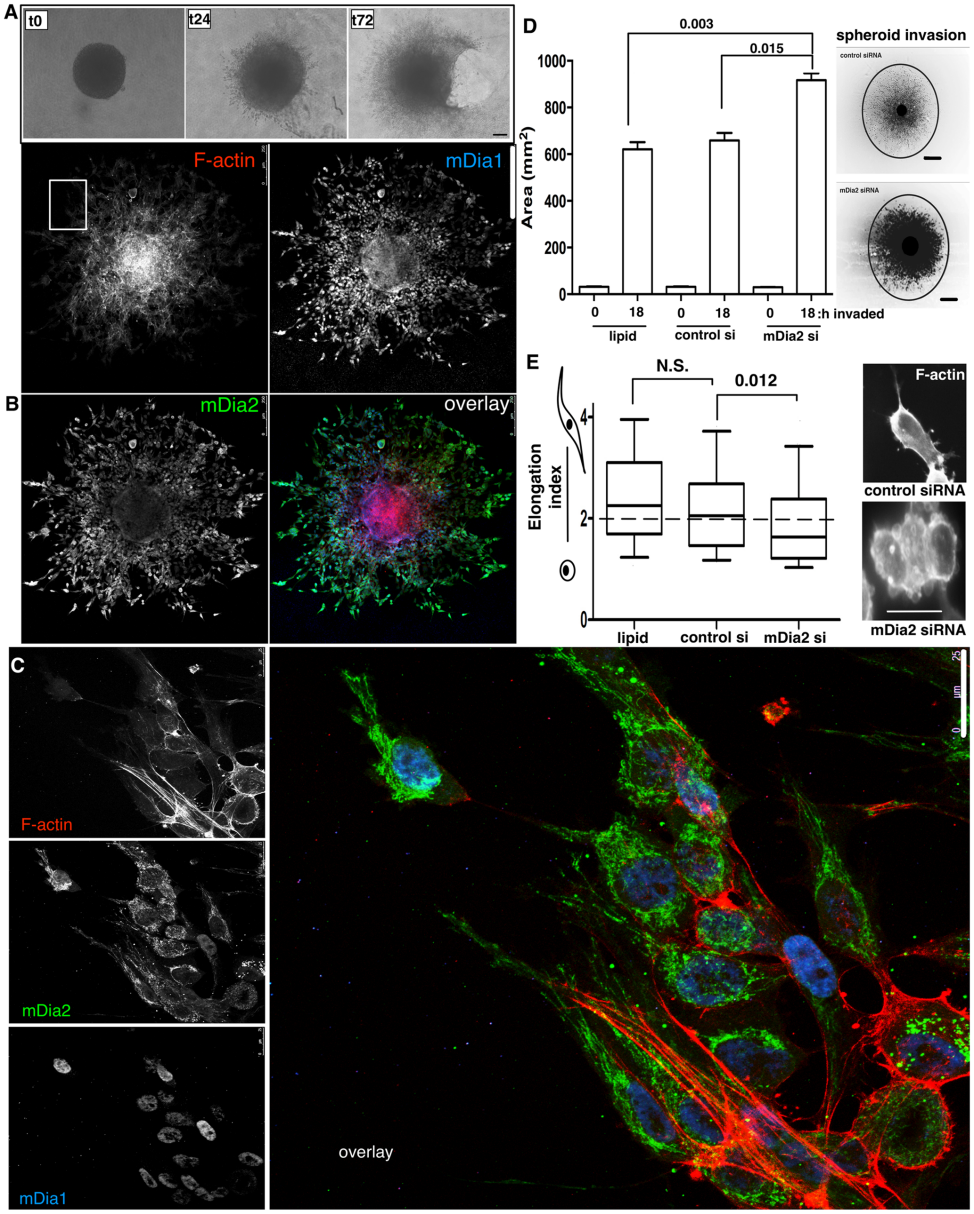
mDia2 depletion drives ES-2 spheroid invasion via amoeboid transitions. A. Spheroids were formed first for 48-I gels, and imaged by brightfield microscopy after the designated times. Bar =  150 µm. B. Spheroids were formed for 72 h, embedded in collagen I gels and allowed to invade for 24 h. Spheroids were fixed and IF performed for mDia1, mDia2 and F-actin. Bar =  100 µm. C. Higher magnification image (63×) of invading cells from the designated ROI in B. mDia1, mDia2, and F-actin were visualized by confocal microscopy. Bar =  25 µm. D. After 56 h post-siRNA treatment, spheroids were formed and embedded in collagen at 104 h post-siRNA treatment. The area for ROI drawn corresponding to 95% of invasive cells was calculated for 0 and 18 h post invasion (122 h post-siRNA treatment). Representative spheroids at 4X are shown stained for phalloidin and images contrast enhanced. Bar =  100 µm. At least 30 spheroids were measured for each condition. p values are shown above the histogram and are relative to invasion at 18 h for either lipid or control treated spheroids. E. Elongation indices were calculated for invasive cells from D. At least 30 invasive cells were measured per condition. Representative phalloidin staining is shown. Bar =  25 µm. EI> 2 (dashed line) is considered mesenchymal cell type. p values are shown above the plot and are relative to invasion at 18 h for either lipid or control treated spheroids. All error bars correspond to SDs from a representative experiment.

To evaluate whether the RhoA/mDia2 axis functions in spheroid invasion, ES-2 spheroids were embedded in collagen at (T0) and allowed to invade through 168 h (Figure 5A). A rapidly advancing front of invasive cells appeared by 24 h, and gel contraction was extensive enough to rip the gels within 72 h. Spheroid invasion was measured by drawing a region of interest around the perimeter of the invasive front, which encompassed at least 95% of invasive cells (as determined by fluorescence labeling). Invasion depended upon Rho GTPases, as the invasive front was markedly reduced in ES-2 spheroids that were grown and embedded in the presence of C3 transferase, relative to vehicle control-treated spheroids (Figure S2, B and C). Since Rho-directed mDia proteins drive invasion in a variety of epithelial-derived cancer cells [26], [29], [30], we visualized the spatial localization of mDia proteins in fixed invasive ES-2 spheroids. mDia2 was distributed in a polarized pattern, with enrichment at the spheroid edges where invasive cells emanated into the collagen (Figure 5B). At higher magnification of the invasive front, mDia2 was dramatically localized to elongated structures resembling tubulated endosomes [53], which extended throughout the long protrusions of the mesenchymal cells. Interestingly, mDia1 was mostly nuclear in invading cells, consistent with previous reports for nuclear activated mDia1 directing SRF-mediated F-actin dynamics within the nucleus [54]. We observed multiple cellular morphologies in invasive cells in collagen, including spherical amoeboid cells (∼40% invading cells), and elongated, polarized mesenchymal cells (∼60% invading cells) migrating in strands (data not shown). Interestingly, invasive spheroids reproducibly (using several primary antibodies of different host and commercial derivations) showed distinct, punctate mDia2 staining within the matrix (Figure 5C, and data not shown). Previously, mDia proteins were detected in pro-migratory OvCa-derived microparticles [55] and mDia2 directed pro-migratory oncosome formation in prostate cancer cells [29]. Biochemical studies are currently underway to characterize the mDia2-enriched extracellular particles present in invading OvCa spheroids.

To evaluate the role of mDia in single cell invasion, we measured the invasive area of embedded mDia1 or mDia2-depleted ES2 spheroids after 0 h (104 h post-knockdown) and 18 h (122 h post-knockdown). Untreated and control siRNA-treated spheroids robustly invaded the collagen matrix within 20 h. However, mDia2-depleted spheroids revealed significantly enhanced invasion relative to controls (Figure 5D and Figure S4C), despite their modestly smaller size upon embedding (Figure 4C). Conversely, mDia1-depleted spheroids invaded to the same extent as control spheroids (Figure S6), indicating that mDia1 does not play a significant role in OvCa spheroid invasion. Because changes in mDia2 expression and/or function associated with amoeboid transitions [27]–[29], we calculated elongation indices (EI) in the invasive cells derived from the spheroids by dividing the long axis of the cell by the short axis; An EI of 2 or more indicates an elongated mesenchymal shape, while an EI of less than 2 indicates a more prominent rounded, amoeboid phenotype. The results indicate that in the spheroids where mDia2 depletion enhanced invasion, the invasive cells were predominantly amoeboid in shape relative to untreated and control siRNA-treated spheroids (Figure 5E).

### ROCK supports both mesenchymal and amoeboid 3D motility programs in invasive ES-2 spheroids

**Figure 6:**
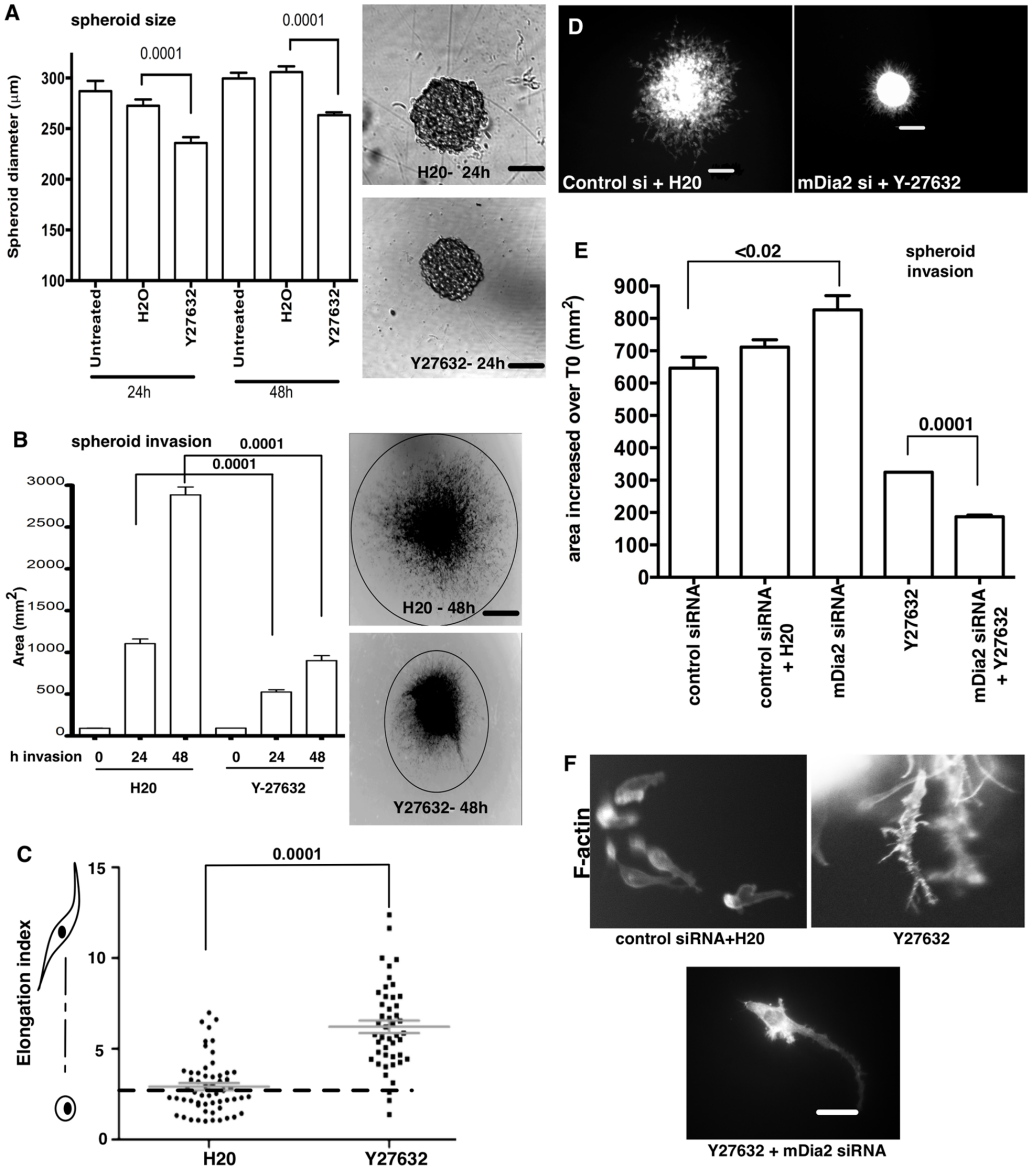
ROCK signaling drives ES-2 spheroid invasion. A. Spheroids were formed in the presence of vehicle or 90 µM Y27632 for the indicated times. Spheroid diameters were then measured for at least 30 spheroids at 10×. Bar =  100 µm. p values are shown above the plot and are control H_2_0-treated spheroids. B. Spheroids were formed for 48 h in the presence of Y27632, and then embedded in collagen-I for 0–48 h in the presence of vehicle or Y27632. Invasive fronts were measured at each time point for at least 30 spheroids per condition. Bar =  400 µm. Representative spheroids at 4× are shown stained for phalloidin and images contrast enhanced. P values are listed above the graph and are relative to control H_2_0-treated spheroids. C. Elongation indices were calculated for invasive cells in the collagen gels shown in B. EI> 2 (dashed line) were considered mesenchymal cell type. At least 30 cells were measured per condition. p values are listed above the chart and are relative to control H_2_0-treated spheroids. D–E. Spheroids were formed in the presence of Y27632; siRNA-treated spheroids were formed at 48 h post-siRNA treatment in the presence of 90 µM Y27632. Spheroids were then embedded in collagen for 24 h Y27632 was maintained in both the gel and media with spheroids through 104 h post-siRNA treatment, and upon embedding for an additional 24 h. Spheroids were fixed and stained for F-actin. Images shown in D are 4× and scale bar =  200 µm. At least 20 spheroids were measured for each condition for E. p values are listed above (E) and are relative to control siRNA-treated spheroids, or Y27632-treated spheroids, as noted. F. Representative 40× phalloidin staining of invasive cells. Bar =  40 µm. All error bars correspond to SDs from a representative experiment.

Amoeboid transitions can involve Rho-directed ROCK signaling to direct contractility [56], [57]. ROCK also antagonizes mDia proteins with respect to maintaining cell-cell junctions in 2D monolayers [18]. However, the interplay between ROCK and mDia proteins for maintaining cell-cell junctions in 3D structures such as OvCa spheroids is unclear. We therefore assessed the role of ROCK activity in spheroid formation. We blocked ROCK activity by treating spheroids with the specific inhibitor, Y27632. Spheroid size was slightly reduced through 48 h of treatment (Figure 6A), suggesting that ROCK/myosin-mediated crosslinking of mDia-regulated F-actin may play a role in spheroid formation. Inhibition of ROCK dramatically suppressed spheroid invasion relative to controls (Figure 6B), consistent with prior studies implicating ROCK in blebbing of colorectal [58] and Caov3 ovarian cancer monolayer cells [59]. A strikingly exaggerated mesenchymal transition predominated in the few cells populating the modest invasive front in cells treated with the ROCK inhibitor (Figure 6C), indicating a requirement for ROCK to effectively direct cellular contractility in both amoeboid and mesenchymal invasion programs.

Finally, we asked whether enhanced invasion upon mDia2 depletion was due to transition from mDia2-driven mesenchymal to ROCK-driven amoeboid invasion. mDia2-depleted spheroids were treated with Y27632 and invasion evaluated after 24 h (Figure 6D–F). As before, both mDia2-kncokdown and Y27632 treatment caused the ES-2 cells to form slightly smaller spheroids relative to controls at T0 (Figure S7). mDia2 depletion also enhanced invasion, whereas ROCK inhibition suppressed invasion (Figure 6D–F). Strikingly, simultaneously inhibiting both pathways completely blocked invasion and total spheroid diameters were comparable to T0 embedded spheroids. Few invasive cells were observed upon combined ROCK and mDia2 inhibition (Figure 6D). This contrasted with the mixed morphology observed in the plethora of control invasive cells. mDia2 depletion drove a predominant amoeboid morphology (Figure 5E). Yet, ROCK inhibition Y27632 alone or combined mDia2/ROCK suppression resulted in highly elongated cells amongst the very few cells that did invade (Figure 6F). This invasive morphology was similar to that observed upon inhibition of Rho GTPase with C3 transferase treatment (Figure S2D), suggesting that RhoA directs migratory plasticity through its effectors ROCK and mDia2.

## Discussion

Much of what we know about tumor cell invasion programs is derived from Boyden-chamber type experiments, in which single cell suspensions are seeded atop layers of matrigel. While informative, these results do not fully address mechanisms driving the rare invasive events initiated from a 3D tumor mass. Our current study dissects the contribution of the F-actin assembly factor mDia2 in invasive transitions using a clinically relevant OvCa spheroid model. Multi-cellular spheroids represent more invasive and tumorigenic OvCa cell populations [3], [4], [10]. Selection for this subset of chemotherapy-resistant OvCa cells could influence disease progression and/or recurrence. Little is known about the molecular underpinnings of OvCa spheroid formation or the cellular cues that trigger single cell invasive egress from spheroids into the underlying peritoneal mesothelium. Our data reveal that mDia2 induces highly adaptable single cell invasion from OvCa spheroids by functioning within a RhoA/ROCK-mediated cytoskeletal signaling network. While single cell invasion from spheroids utilizes both mesenchymal and amoeboid motility, mDia2 depletion specifically impacts spheroid organization/integrity so that amoeboid invasion predominates. Our results also show that suppression of ROCK inhibits spheroid single cell motility, by inducing exaggerated cellular elongation. Finally, we showed that the amoeboid program induced through mDia2 depletion is dependent upon Rho-mediated activation of ROCK, as spheroid invasion is completely abrogated upon concurrent mDia2 and ROCK inhibition. These data suggest that tightly regulated interplay and interdependence between ROCK and mDia2 signaling dictates the invasive capacities of motile spheroid-derived OvCa cells.

Studies firmly place mDia proteins in the queue of cytoskeleton reorganizing proteins driving tumor progression. Loss of *DIAPH3*, which encodes the mDia2 protein, occurs in primary and metastatic prostate cancer tumors, hepatocarcinoma, breast carcinoma, and in an OvCa progression model [10], [29], [30], [44]. In prostate and ovarian cancer studies, *DIAPH3* was lost steadily from early through late stage disease models, and was accompanied by a disorganized cytoskeleton depleted of F-actin and a more aggressive phenotype [29], [44]. We, along with others, have demonstrated that loss of mDia2 drives amoeboid motility [28]–[31]. mDia2 clearly directs plasticity in motility programs: That is, inhibition of mDia2 function promotes amoeboid motility through disruption of cortical F-actin assembly to induce bleb extrusion, while activation of mDia2 promotes assembly of F-actin rich pro-migratory structures such as lamellae, invadopodia and filopodia [26], [46], [52], [60]. Therefore, when considering mDia2 as an anti-motility therapeutic target for tumor cells, inhibition is predicted to be counter-therapeutic in some disease models.

Loss of *DIAPH1,* which encodes mDia1 protein, is associated with myelodysplastic syndrome [42] and defects in T cell and neutrophil trafficking/polarization [32], [34], [35]. mDia1 is also significantly depleted in a cell-culture model of OvCa progression [10], although it is concentrated in pro-migratory microparticles shed from ovarian carcinomas [55]. In the present study, we show that mDia1 depletion affects neither ES-2 spheroid formation nor spheroid invasion (Figures 4 and S6). Interestingly, mDia1 loss was previously shown to slightly diminish RhoA and ROCK activity, the latter of which could favor mDia2-mediated F-actin dynamics [38], [61], [62]. In our system, the highly polarized localization and the temporal regulation of mDia1 may contribute to regulation of F-actin dynamics in migrating OvCa cells. In addition, mDia1 may support the nuclear actin network-as suggested by the extensive nuclear localization of mDia1 in Figure 5C and in [54]). Nevertheless, we show here that mDia2 is the predominant formin involved in mediating 3D OvCa spheroid organization and invasive transitions.

While targeting either mDia2 or ROCK led to slightly smaller spheroids, spheroid architecture was affected only upon mDia2 suppression (Figures 4, S5, respectively). This suggests that mDia2 maintains the integrity and cohesion of compacted spheroids. The individual roles of ROCK and mDia and the functional implications of their potential interplay for regulation of cell-cell junctions in epithelial cells are complex. ROCK and mDia have opposing effects in maintaining/stabilizing cell-cell junctional integrity. High RhoA-GTP levels led to ROCK-dependent junctional destabilization through generation of contractile force. However, moderate RhoA activity favors mDia-directed AJ stabilization [18]. Further, ROCK cooperates with mDia to drive migration via actinomyosin-based contractility. High levels of RhoA-GTP activated both mDia and ROCK, while lower levels preferentially targets mDia1 [63]. This effect is potentially explained by the finding that the K_d_ for mDia1 binding to RhoA-GTP is lower than the K_d_ for ROCK binding to RhoA-GTP [64], [65]. In invasive hepatocyte spheroids, RhoA activation is tightly regulated both spatially and temporally, and is inversely correlated with cadherin engagement within the cell-cell junctions (*i.e.,* high cadherin: low activated-RhoA) [50]. In the latter study, it was suggested that RhoA activation was a consequence of to disruptions of cadherins in cell-cell attachments. This potentially allowed RhoA to interact with effectors (*i.e.,* ROCK, mDia) that promote single cellular migration/invasion. Our data reveal increases in RhoA-GTP as spheroids mature (Figure 3C). Indeed, ES-2 spheroids caused contraction of 3D collagen-I gels, which is indicative of high actinomyosin forces and, by inference, Rho/ROCK activity [4]. Collectively these data suggest that within the spheroid a balance between mDia2 and ROCK activity exists, coordinated by levels of RhoA-GTP as well as the spatial and temporal localization of RhoA-GTP [62] and its effectors. The underlying spheroid cell-cell junctions are potentially supported by mDia2-mediated F-actin dynamics, under conditions of lower RhoA-GTP levels. As RhoA-GTP levels increase during spheroid maturation and upon engaging the extracellular matrix, ROCK signaling may ultimately prevail to disrupt AJ, and destabilize the spheroid architecture. mDia2 suppression (Figures 5–6) potentially promotes invasion transitions due to default RhoA-mediated ROCK activation (and junctional destabilization). Experiments are currently in progress to test this hypothesis.

While ROCK inhibition failed to affect spheroid formation, it dramatically reduced spheroid invasion within collagen (Figure 6). Both amoeboid and mesenchymal morphologies were present in control spheroids, yet ROCK inhibition significantly blocked spheroid invasion. The few remaining invasive cells had dramatically increased EI indicative of defects in retraction, as previously demonstrated [66]–[68]. Our data supports the notion that activated ROCK promotes both mesenchymal and amoeboid motility. Inhibition of ROCK therefore has a dual effect: 1., suppressing actinomyosin-based contractility required for initiation and retraction of amoeboid blebs [69]–[72], and 2., facilitating extension/retraction of mesenchymal cellular protrusions [73]. It is unclear from our 3D experiments whether amoeboid or mesenchymal single cell invasion represents a more efficient mode of invasion, as judged by distance travelled, velocities, etc. Experiments addressing this question are currently underway.

Our data shed light on a possible mechanism whereby modulating the RhoA/mDia/ROCK axis induces invasive transitions in OvCa spheroids. The work of Brugge and colleagues recently revealed that OVCA433 spheroids generated actinomyosin contractility to clear the underlying mesothelia and drive OvCa dissemination [74]. The activation mechanism of myosin contractility has not been explored. The enhanced RhoA-GTP levels present in mature spheroids may act as a driver for forces inducing single cell transmesothelial migration and/or clearance. It is tempting to speculate that induction of Rho/ROCK/mDia2-dependent amoeboid motility promotes efficient single cell transmesothelial migration. Similarly the Rho/ROCK axis could generate actinomyosin-based force to induce mesenchymal invasion. It will be interesting to determine if targeting ROCK in the spheroid-mesothelial invasion model inhibits mesothelial clearance, thereby disrupting both mesenchymal and amoeboid single cell invasion.

Finally, we have shown that mDia2 depletion effectively induces amoeboid invasive transitions in OvCa spheroids (Figure 5). It is unclear what physiological triggers, outside of loss of mDia2, could promote such invasive transitions *in vivo*. We previously showed that CXCL12 promotes amoeboid transitions [28] through the formation of a complex between mDia2 and its functional inhibitor, DIP, in MDA-MB-231 breast cancer cells. A role for CXCL12 and its receptor, CXCR4, in OvCa progression, peritoneal dissemination, and metastasis has also been established [75]–[80]. Elevated levels of mesothelium-derived CXCL12 have been detected in patient ascites and expression of CXCR4 in OvCa cells correlated with a poorer prognosis and reduced survival [76]. A potential role for CXCR4 in driving OvCa metastasis is further supported by the finding that treatment of xenografted ES-2 cells with a CXCR4 antagonist reduced peritoneal dissemination of the tumor cells[75]. In light of these and our current studies, it would be interesting to evaluate whether CXCL12 promotes invasive transitions and peritoneal dissemination through functional inhibition of mDia2 in OvCa spheroids.

## Conclusions

Our findings indicate that multiple Rho GTPase effectors must be suppressed to fully block egress of invasive cells from 3D ovarian cancer spheroids. Furthermore, tightly regulated interplay and interdependence between ROCK and mDia2 signaling pathways dictates the invasive capacities and the type of invasion program utilized by motile spheroid-derived ovarian cancer cells. Loss of the gene encoding mDia2, *DRF3,* has been linked to cancer progression and metastasis. Our results set the stage for a broader understanding of the molecular mechanisms involved in mDia2-dependent dissemination of invasive cells tumor cell from primary tumor lesions, potentially in a variety of epithelial cancers.

## Methods

### Cell lines, chemicals, plasmids and transfections

ES-2, SKOV3, OVCAR3 cells were from ATCC. OVCAR4 was from the National Cancer Institute's Tumor Repository. OVCA cell lines OVCA 420 and OVCA 429 are cell lines previously established and derived from patients with late-stage serous ovarian adenocarcinomas [81]. The OVCA194 cell line was were previously described [82]. The OVCAR4, OVCA429, OVCA420 and OVCA194 were kind gifts from Drs. Deborah Vestal and William Maltese. OVCA429, SKOV3, OVCAR4, OVCA420, OVCA194, and ES-2 cells were grown in RPMI-1640 containing 10% (v/v) FBS and 100 µg streptomycin in a 37°C incubator with 5% CO_2_. OVCAR3 cells were grown in RPMI-1640 containing 20% FBS and 100 µg streptomycin. The constructs encoding EYFP, EYFP-mDia2, EYFP-mDia1, EYFP-FH2ΔN, and EYFP-mDia2 M1041A were kind gifts from Dr. Art S. Alberts. Y27632 was from Sigma. SMIFH2 was from EMD Biochemicals.

OVCA429 cells were transfected with the preceding expression constructs for 48 h using Fugene (Promega), per the manufacturer's protocol. For siRNA-mediated suppression of mDia1or mDia2, 25–100 nM smart pool or individual siRNAs targeted against the human sequences were obtained from Dharmacon and transfected into adherent cells using the Dharmafect-1 reagent (Thermoscientific). Parallel cultures were transfected with control siRNAs.

### Adhesion, Migration and Invasion Assays

Cells were loaded with 4 µg/ml Calcein AM-FITC (Invitrogen) for 15 min at 37°C prior to adhesion assays. Cells (1×10^4^) in serum-free media (SFM) were added to 96-well plates coated overnight with 10 µg/ml collagen-I (BD Biosciences) or BSA (Sigma). Cells were incubated for 45 min at 37°C, after which wells were aspirated and washed five times with PBS/0.5% BSA, rotating the plate 90° with each wash. Adherent cells were detected in a fluorescent plate reader (485/538 nm excitation/emission), with total adherent cells extrapolated from a standard curve derived from a known number of cells. Adhesion assays were performed in triplicate and the experiment was repeated thrice. Standard deviations (SDs) were expressed from triplicate wells from a representative experiment.

For migration, cells were serum-starved overnight and loaded with Calcein AM-FITC as above. 1×10^5^ cells in SFM were added to the upper well of 8 µm pore-size Fluoroblok inserts (Falcon) coated overnight with 10 µg/ml collagen-I (BD Biosciences). SFM or 20% FBS was added to the lower well. Cells were incubated for 5 h at 37°C prior to enumerating migrating cells in a fluorescent plate reader. Migration assays were performed in triplicate and the experiment repeated thrice. SDs were expressed from triplicate wells from a representative experiment.

Invasion assays were performed as previously described [28]. Briefly, 50,000 Calcein AM-FITC labeled cells were seeded upon 2 mg/ml collagen gels previously polymerized within the upper well of 8 µm pore inserts. Invasion assays were performed in triplicate and the experiment was repeated thrice for 24 h at 37°C in response to either SFM or 20% FBS gradients. SDs were expressed from triplicate wells from a representative experiment.

### Microscopy

Confocal imaging of live and fixed cells was performed using a Leica Microsystems TCS SP5 multiphoton laser-scanning confocal microscope system with a 8000 Hz resonant scanner and environmental controls for maintaining 37°C, humidity and 5% CO_2_, and laser lines of 488, 514, 633 and 710–990 nm from a tunable Ti-Sapphire MP laser. Hardware is controlled from the Leica AFM acquisition/analytical software suite. MetaMorph software suite (Molecular Devices) was used for data analysis. Dunn chamber acquisition was performed using a Leica 10X HC PL APO CS Dry UV 0.40NA objective lens. Confocal spheroid imaging was performed using a Leica 20X HCX PL APO CS Dry UV 0.70NA objective lens. Spheroids processed for immunofluorescence were stored and imaged in RPMI.

Immunostained coverslips and brightfield documentation of spheroid formation were acquired using an IX81 inverted microscope (Olympus) equipped with YFP, TRITC and CY5 filters. Acquisition and analysis were performed using MetaMorph software suite. 2D cell images were acquired with an Olympus 40X UPlan Apo 1.00 oil lens; spheroid images for measuring size and invasion were acquired with either an Olympus 10X UPlanFL 0.30NA objective lens, or Olympus 4x UPlanFL 0.13NA objective lens, respectively. Coverslips and spheroids were mounted using Fluoromount-G mounting media (Southern Biological) and RPMI, respectively.

### Dunn Chemotactic Migration Assay

Dunn chambers (Hawksley) were set up as described [83], [84]. Coverslips were coated with 10 µg/ml collagen-I (BD Biosciences) overnight. Cells were loaded with Calcein AM-Red Orange (Invitrogen) for 15 min at 37°C. Cells (2.5×10^4^) were plated and allowed to adhere overnight. The inner well of the chamber was filled with SFM. The outer well was exchanged with medium containing 20% (v/v) serum. The chamber was maintained at 37°C with humidity on the microscope stage. The cells located over the bridge were imaged every 15 min for 5 h.

### Spheroid formation and collagen embedding

Spheroids were formed by centrifugation of poly-HEMA coated plates as described [85]. Briefly, 96-well plates were coated with 0.5% poly-HEMA and dried at 37°C. Cells were detached using Versene (Invitrogen) and resuspended in RPMI with 10% FBS and 2.5% of 15 µl/ml matrigel (BD Biosciences). 500 cells per well were pelleted by centrifugation at 1000×g for 10 min. Unless specified, spheroids were formed for 72 h before use. Collagen-I was prepared at 2 mg/ml; a thin layer was partially polymerized in 8-well chamberglass (Lab-Tek) before adding spheroids, and excess media removed before adding the top layer of collagen. Gels were polymerized for 45 min before adding media or fixing, as described [4].

### 2D Immunofluorescence (IF)

Cells imaged by IF were processed as described [28]. Briefly, cells were plated upon sterilized coverslips overnight, fixed in 4% paraformaldehyde for 20 min, permeabilized with 0.2% Triton X-100 for 20 min and stained with antibodies against mDia2 and mDia1 (Proteintech) overnight at 4°C followed by incubation with Alexa-Fluor secondary antibodies (Invitrogen) for 4 h at 37°C. Alexa-Fluor-conjugated phalloidin (Invitrogen) was used to visualize the F-actin architecture. DAPI (Invitrogen) was used for nuclear staining.

### 3D Immunofluorescence and Invasion of Spheroids

Embedded spheroids were stained as described, with minor changes [86]. Briefly, invasive spheroids were fixed in 4% paraformaldehyde for 20 min at RT, washed in PBS, and permeabilized with 0.5% Triton X-100 for 60 min at RT. Spheroids were washed with 100 mM glycine before blocking. Primary antibodies were incubated at 4°C overnight, and incubated with Alexa-Fluor secondary antibodies (Invitrogen) and Alexa Fluor 488 phalloidin (Invitrogen) at 37°C for 3 h or at 4°C overnight. The specimen was washed, DAPI was added for 20 min at RT, washed once in PBS then coated in Fluoromount-G (Southern Biotech). Images were collected using an Olympus IX-81 microscope. Invasive area was calculated using Metamorph software, by drawing a region of interest (ROI) upon embedding and after invasion around the spheroid invasive front. The R0I area was selected and drawn to encompass the spherical perimeter of invasive cells and validated with Metamorph software to contain at least 95% of fluorescent pixels corresponding to phalloidin staining.

### RhoA G-LISA

RhoA G-LISA (Cytoskeleton, Inc) was performed according to the manufacture's specifications. Briefly, monolayer cells were grown to confluence before being stimulated with 10 µM 1-Oleoyl Lysophosphatidic acid LPA (Cayman Chemical) for 5 minutes. Cells were collected using the lysis buffer from the kit. A minimum of 200 spheroids were collected at 48, 72, 96, and 120hr. Spheroids were collected on ice, centrifuged briefly to pellet. Lysis buffer was added and the spheroids were dissociated before starting the assay. Each condition was testing in triplicate and the experiment was performed thrice. Standard deviations (SD) were expressed from at least triplicate wells for a representative experiment. Luminescence was measured on the SpectraMax5 (Molecular Devices).

### Statistical Analysis

One-tailed Students t-tests were performed with a 95% confidence value. p values of 0.05 or less were considered statistically significant. All error bars are expressed as standard deviations (SDs) from representative experiments, unless otherwise noted. Graphs and statistics were generated in GraphPad Prism software.

## Supporting Information

Figure S1 **Spheroid formation in OvCa cells.** The formation of spheroids from single cell suspensions was documented after 24 h by brightfield microscopy. Bar = 50 µm.
(TIF)

Figure S2 **Requirement for Rho GTPases in ES-2 spheroid compaction and invasion.** A. Spheroids were formed for 48 h from 500 cells in the presence of H_2_0 vehicle, 0.5, 1.0 or 2.0 µg/ml. Scale bar = 400 µm. B., Spheroids were then embedded in collagen gels and T0 measurements taken. Spheroids were allowed to invade for 18 h. Scale bar =  1000 µm. C., ROI were drawn to measure the invasive area, encompassing at least 95% of cells, at T0 and T18. D., After 18 h, spheroids were fixed and stained with phalloidin, and invasive edges images by fluorescent epifluorescent microscopy. Arrows indicate highly elongated cells emanating from the limited invasive edge. Images were acquired using a 10X objective. Scale bar =  1000 µm.
(TIF)

Figure S3 **Levels of total mDia2, mDia1 and RhoA in monolayers and spheroids.** Spheroids were formed for the indicated times. Cell lysates were collected from pooled spheroids or monolayers and Western blots were performed for mDia2 (A), mDia1 (B) or RhoA (C), along with tubulin as a loading control. Densitometry was performed on the resulting blots (Figure 3) and ratios of mDia1,mDia2, or RhoA:tubulin were determined. Values shown were normalized by setting monolayer values to 100 percent.
(TIF)

Figure S4 **mDia2 depletion via both pooled and single siRNAs influence spheroid formation.** A. Time course analysis of mDia2 depletion in monolayers treated with individual siRNA (mDia2 06, 08) or pooled mDia2 siRNA. Lysates were prepared at the designated times and western blotted for mDia2 or tubulin (loading control). B. ES-2 spheroids were formed from cells depleted for 72 h with individual siRNA (mDia2 06, 08) or pooled siRNA. Spheroid diameters were measured 48 h post spheroid formation. At least 12 spheroids were measured for each condition. p values are listed above the chart and are relative to control siRNA-treated spheroids. C. Spheroids from B were embedded in collagen gels and allowed to invade for 18hrs, after which spheroids were fixed, stained with phalloidin and imaged. ROI were drawn to measure the invasive area, encompassing at least 95% of cells, at T0 and T18. p values are listed above the chart and are relative to control siRNA-treated spheroids. Error bars correspond to SD for a representative experiment.
(TIF)

Figure S5 **Functional mDia inhibition promotes disorganized spheroid formation.** ES-2 spheroids were formed in the presence of 10 µM SMIFH2. Images were acquired by brightfield microscopy after 48 h formation. Bar = 50 µm.
(TIF)

Figure S6 **mDia1 depletion does not alter spheroid single cell invasion.** mDia1 or control GAPDH were depleted in ES-2 monolayers. Spheroids were formed for 48 h, embedded and invaded for 24 h. p values are shown above the chart and are relative to untreated spheroids. Error bars correspond to SD for a representative experiment
(TIF)

Figure S7 **Spheroid diameter measurements upon embedding in response to combined ROCK/mDia2 inhibition.** Spheroids were formed in the presence of Y27632; siRNA-treated spheroids were formed at 48 h post-siRNA treatment in the presence of 90 µM Y27632. Spheroids were then embedded in collagen and T0 measurements taken. At least 20 spheroids were measured for each condition. p<0.01, relative to control si + vehicle control spheroids. Error bars correspond to SDs for a representative experiment.
(TIF)
