# Supplementary figures and images for: An mDia2/ROCK Signaling Axis Regulates Invasive Egress from Epithelial Ovarian Cancer Spheroids
Source: PLoS One. 2014 Feb 28;9(2):e90371. doi: 10.1371/journal.pone.0090371 (PMC3938721; doi:10.1371/journal.pone.0090371)

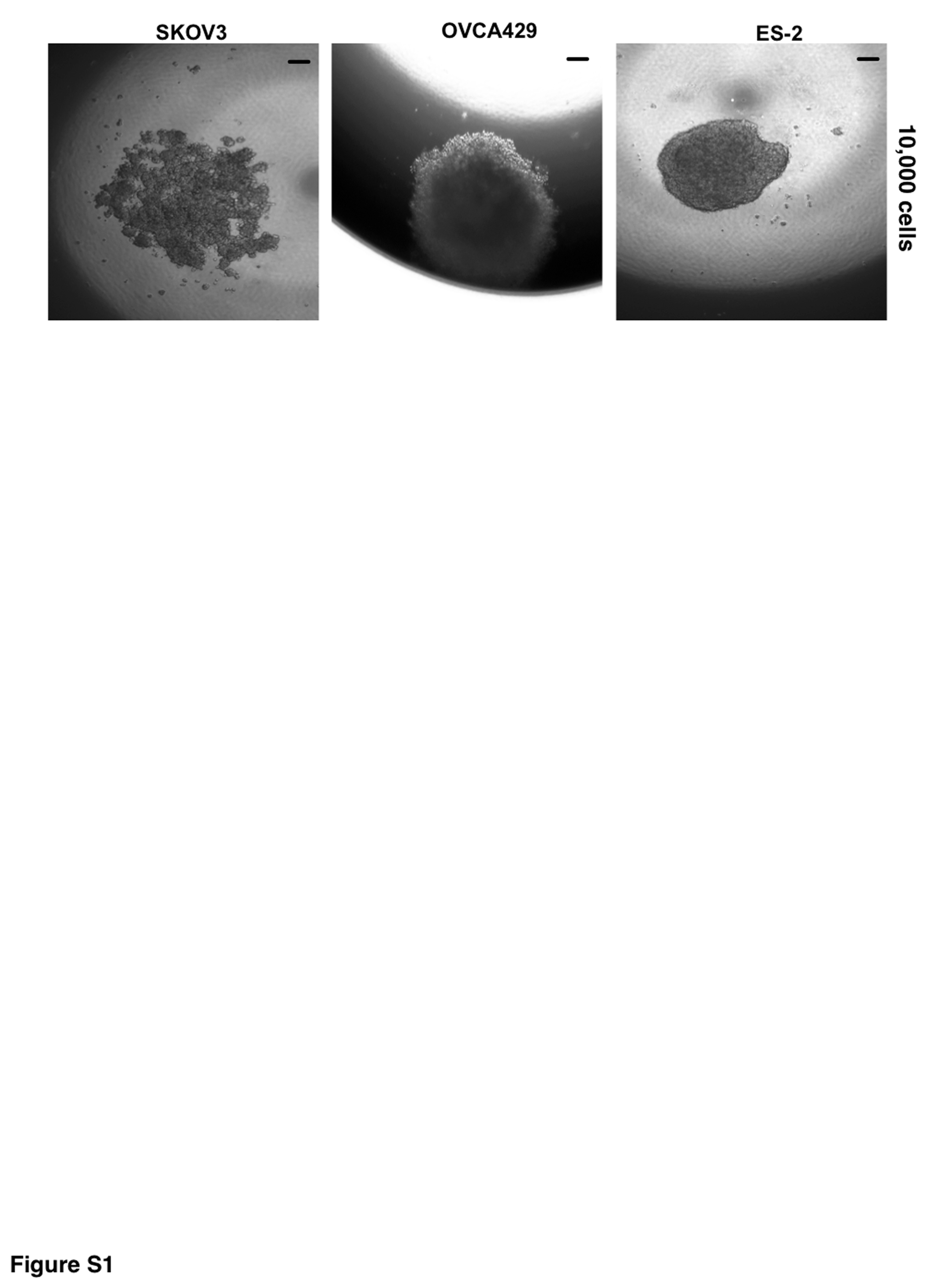

Supplement: Figure S1 — Spheroid formation in OvCa cells. The formation of spheroids from single cell suspensions was documented after 24 h by brightfield microscopy. Bar = 50 µm. (TIF) [file pone.0090371.s001.tif]

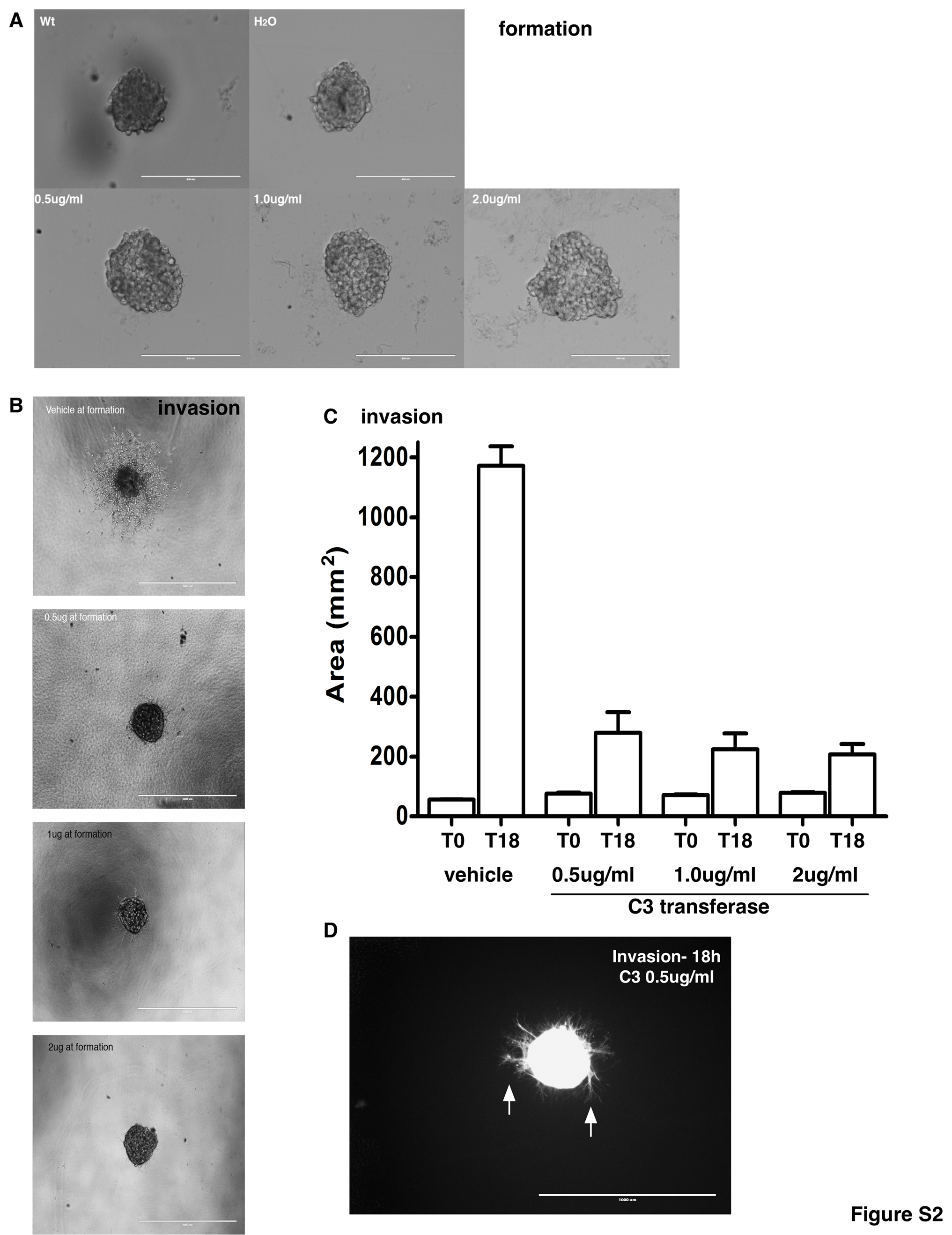

Supplement: Figure S2 — Requirement for Rho GTPases in ES-2 spheroid compaction and invasion. A. Spheroids were formed for 48 h from 500 cells in the presence of H20 vehicle, 0.5, 1.0 or 2.0 µg/ml. Scale bar = 400 µm. B., Spheroids were then embedded in collagen gels and T0 measurements taken. Spheroids were allowed to invade for 18 h. Scale bar = 1000 µm. C., ROI were drawn to measure the invasive area, encompassing at least 95% of cells, at T0 and T18. D., After 18 h, spheroids were fixed and stained with phalloidin, and invasive edges images by fluorescent epifluorescent microscopy. Arrows indicate highly elongated cells emanating from the limited invasive edge. Images were acquired using a 10X objective. Scale bar = 1000 µm. (TIF) [file pone.0090371.s002.tif]

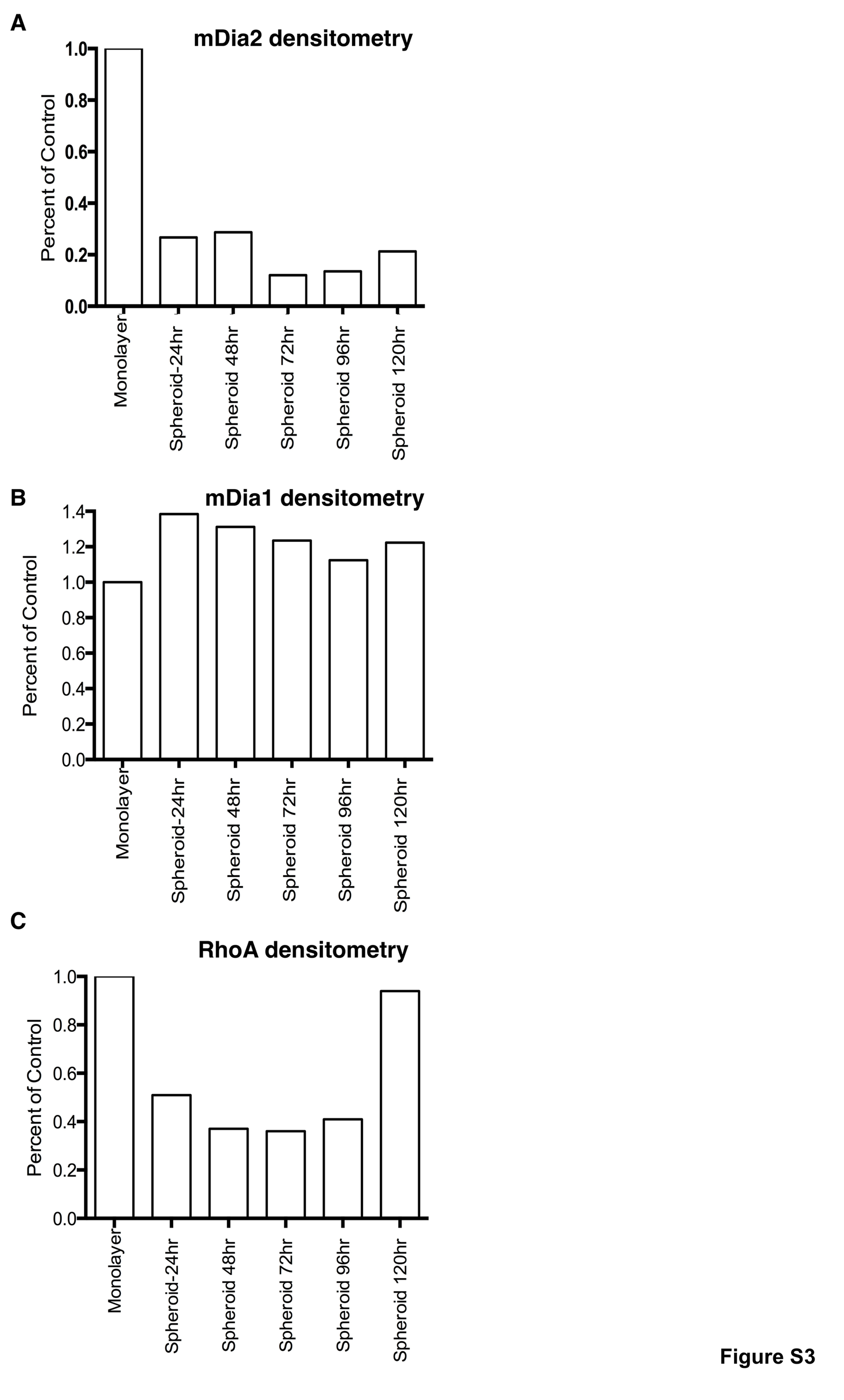

Supplement: Figure S3 — Levels of total mDia2, mDia1 and RhoA in monolayers and spheroids. Spheroids were formed for the indicated times. Cell lysates were collected from pooled spheroids or monolayers and Western blots were performed for mDia2 (A), mDia1 (B) or RhoA (C), along with tubulin as a loading control. Densitometry was performed on the resulting blots (Figure 3) and ratios of mDia1,mDia2, or RhoA:tubulin were determined. Values shown were normalized by setting monolayer values to 100 percent. (TIF) [file pone.0090371.s003.tif]

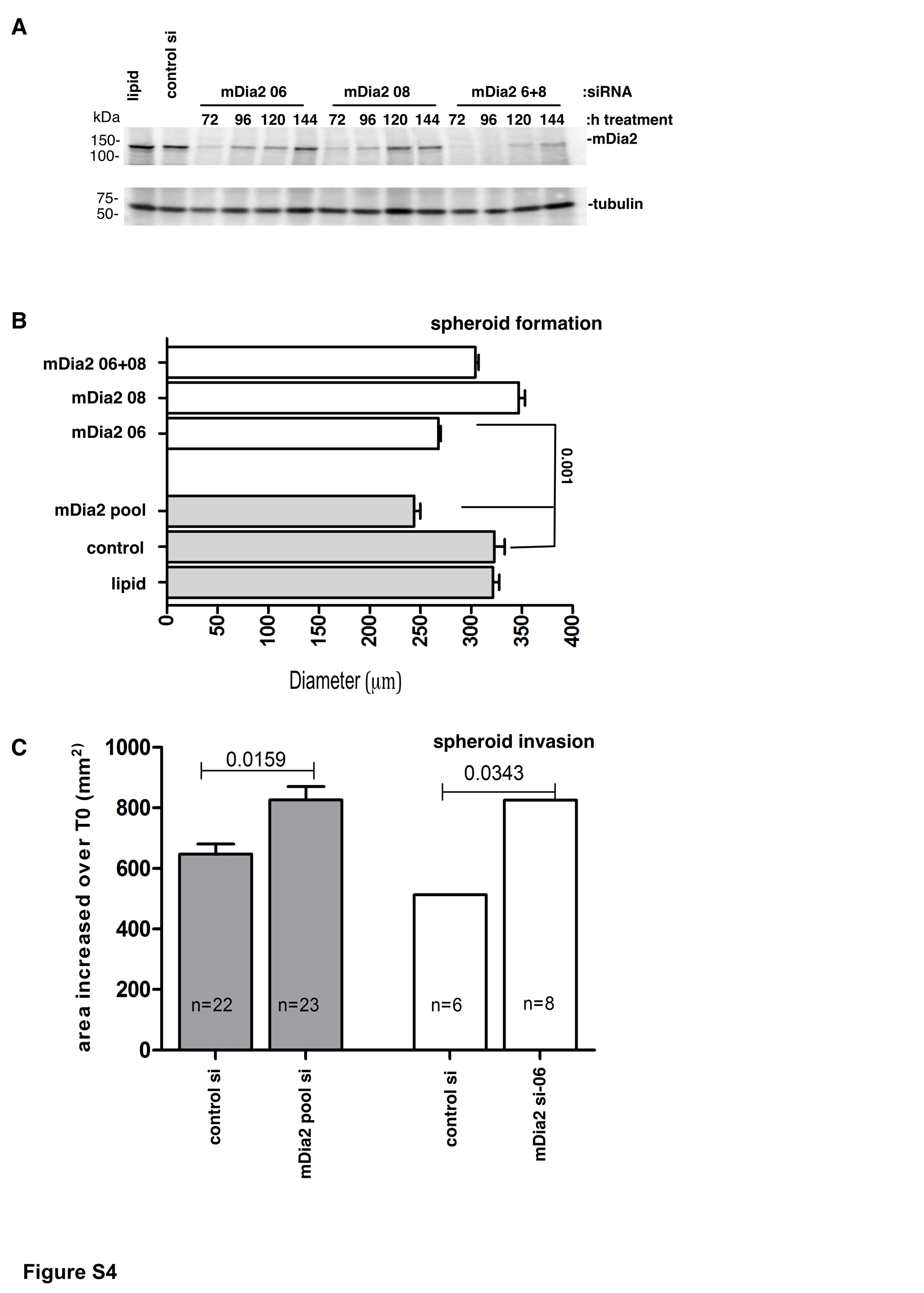

Supplement: Figure S4 — mDia2 depletion via both pooled and single siRNAs influence spheroid formation. A. Time course analysis of mDia2 depletion in monolayers treated with individual siRNA (mDia2 06, 08) or pooled mDia2 siRNA. Lysates were prepared at the designated times and western blotted for mDia2 or tubulin (loading control). B. ES-2 spheroids were formed from cells depleted for 72 h with individual siRNA (mDia2 06, 08) or pooled siRNA. Spheroid diameters were measured 48 h post spheroid formation. At least 12 spheroids were measured for each condition. p values are listed above the chart and are relative to control siRNA-treated spheroids. C. Spheroids from B were embedded in collagen gels and allowed to invade for 18hrs, after which spheroids were fixed, stained with phalloidin and imaged. ROI were drawn to measure the invasive area, encompassing at least 95% of cells, at T0 and T18. p values are listed above the chart and are relative to control siRNA-treated spheroids. Error bars correspond to SD for a representative experiment. (TIF) [file pone.0090371.s004.tif]

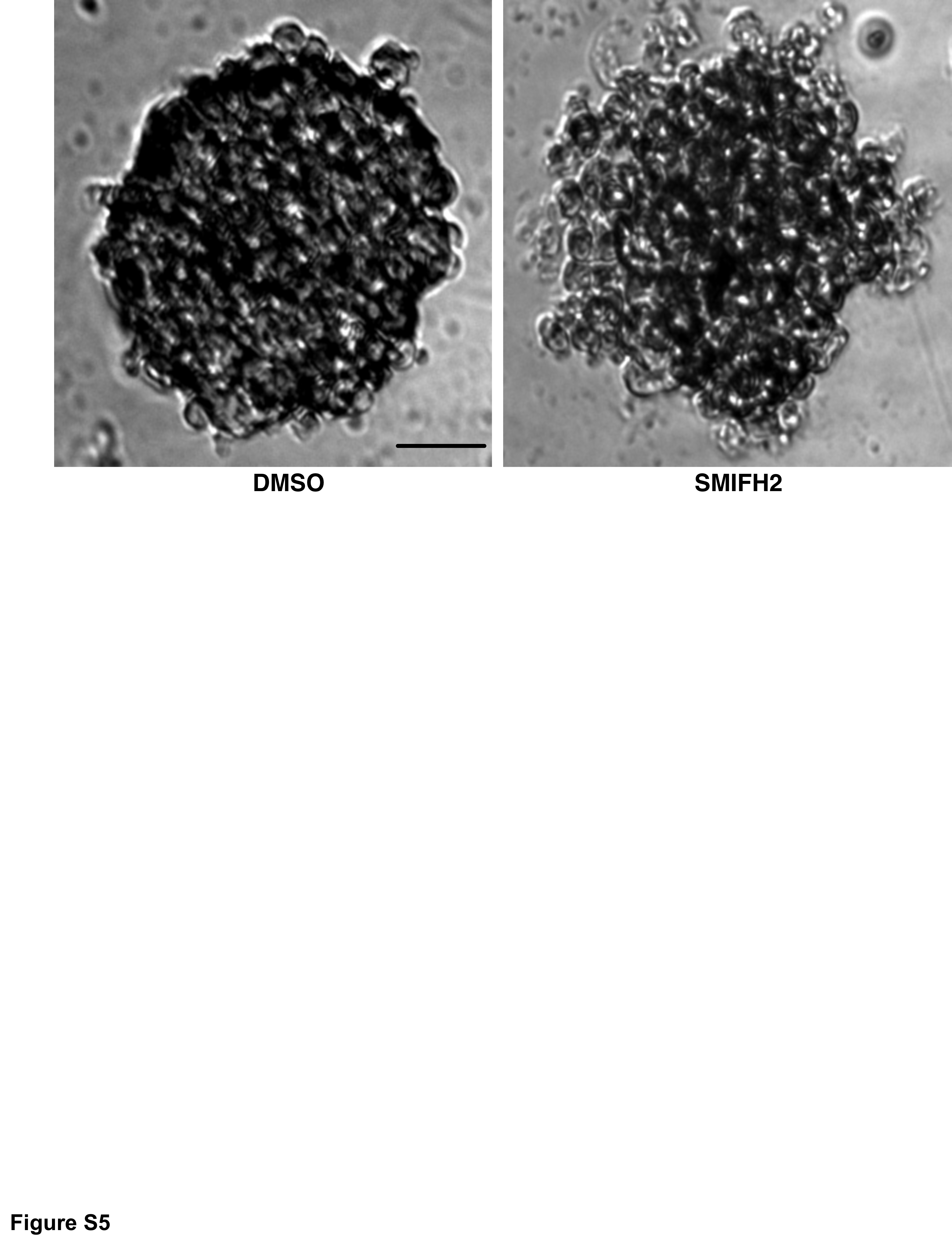

Supplement: Figure S5 — Functional mDia inhibition promotes disorganized spheroid formation. ES-2 spheroids were formed in the presence of 10 µM SMIFH2. Images were acquired by brightfield microscopy after 48 h formation. Bar = 50 µm. (TIF) [file pone.0090371.s005.tif]

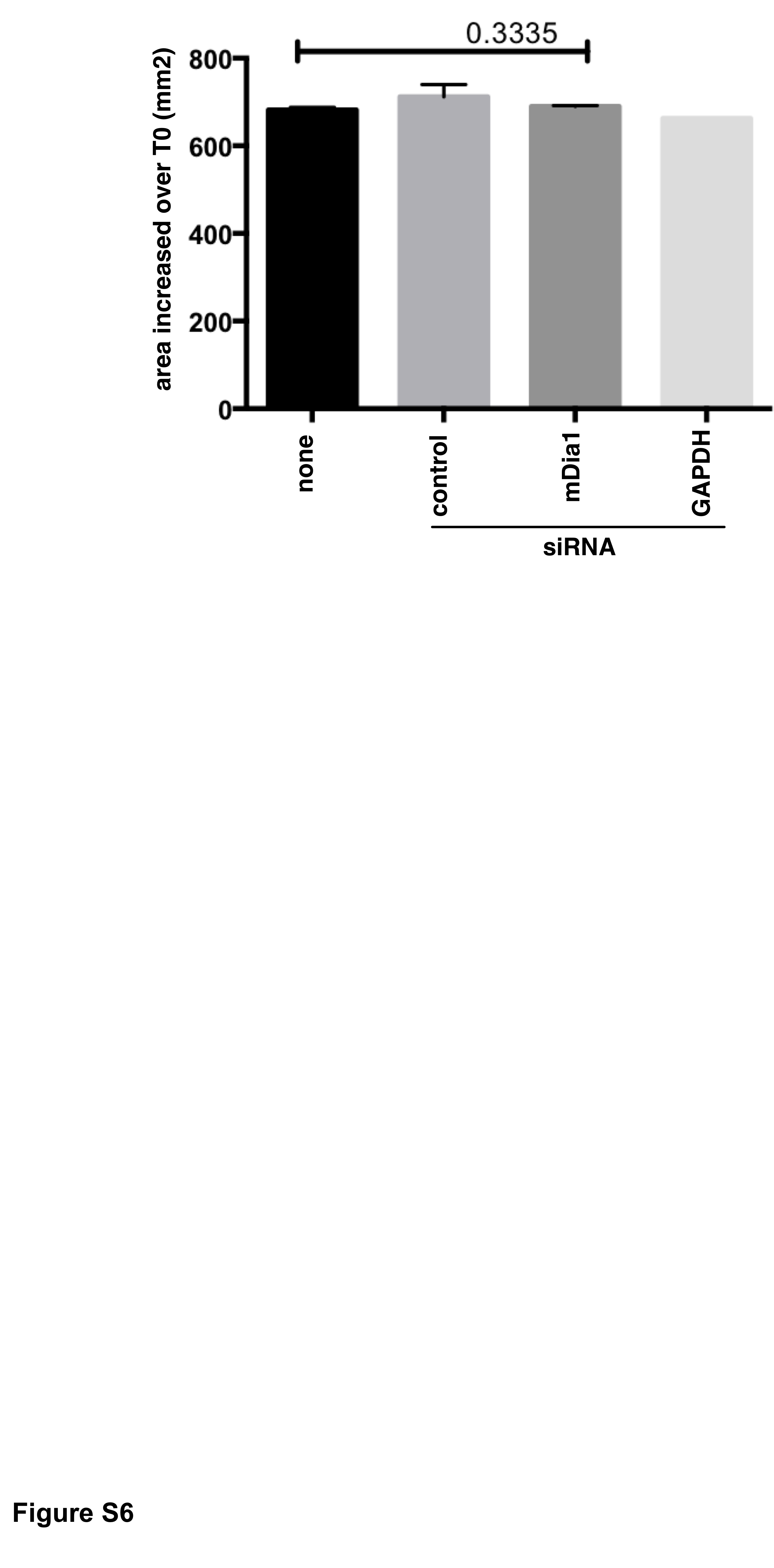

Supplement: Figure S6 — mDia1 depletion does not alter spheroid single cell invasion. mDia1 or control GAPDH were depleted in ES-2 monolayers. Spheroids were formed for 48 h, embedded and invaded for 24 h. p values are shown above the chart and are relative to untreated spheroids. Error bars correspond to SD for a representative experiment (TIF) [file pone.0090371.s006.tif]

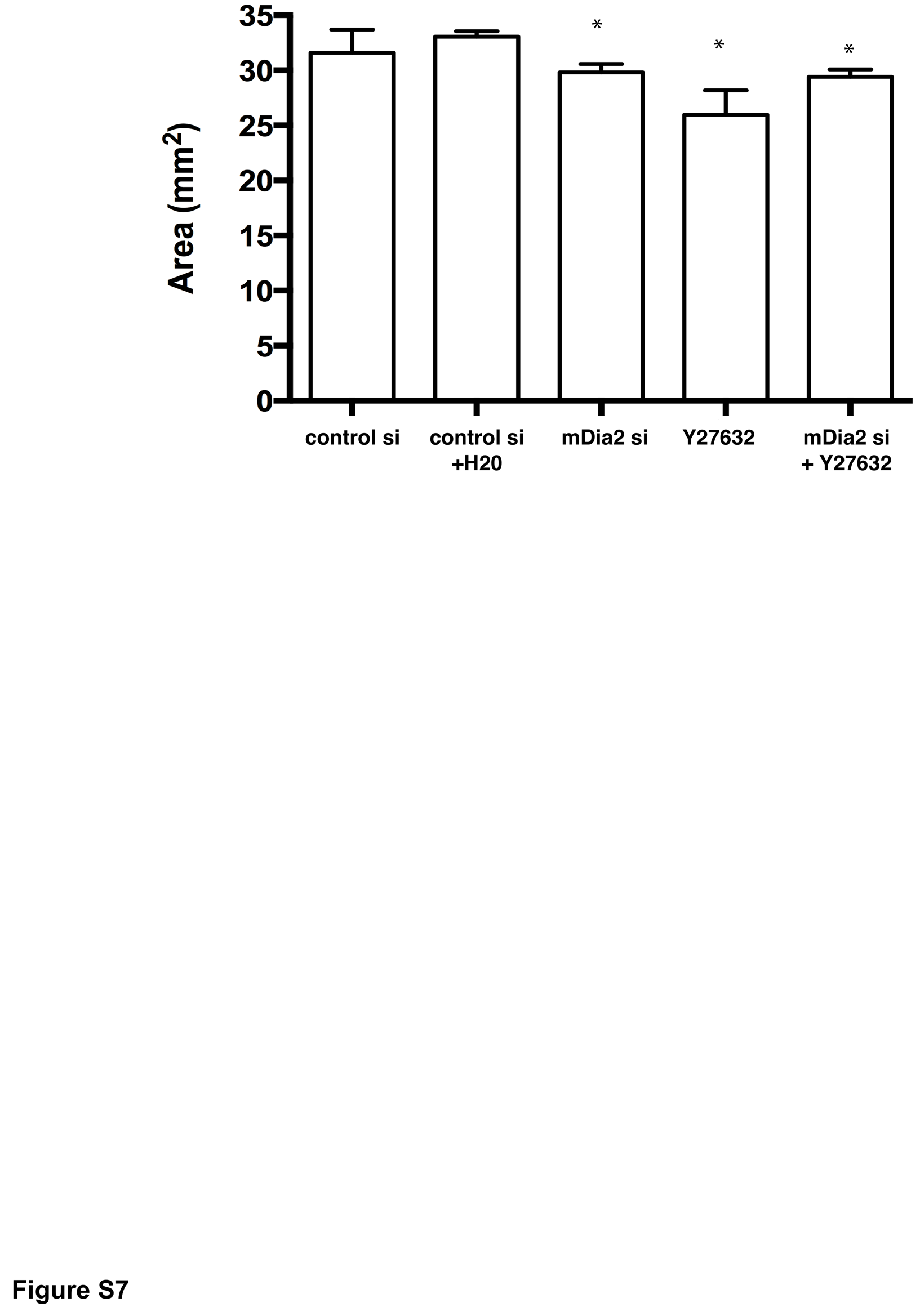

Supplement: Figure S7 — Spheroid diameter measurements upon embedding in response to combined ROCK/mDia2 inhibition. Spheroids were formed in the presence of Y27632; siRNA-treated spheroids were formed at 48 h post-siRNA treatment in the presence of 90 µM Y27632. Spheroids were then embedded in collagen and T0 measurements taken. At least 20 spheroids were measured for each condition. p<0.01, relative to control si + vehicle control spheroids. Error bars correspond to SDs for a representative experiment. (TIF) [file pone.0090371.s007.tif]
